# Development of a web-based care networking system to support visiting healthcare professionals in the community

**DOI:** 10.1186/s12913-023-10434-6

**Published:** 2023-12-16

**Authors:** Jakyung Lee, Susan Park, Mi-hee Cho, Ji-Won Kang, Minkyoung Kim, Suhyeon Choi, Seok-gyu Kim, Ji-hee Choi, Keumhee Han, Chang-O Kim, Il-Chul Moon, Moon Choi, Soong-nang Jang

**Affiliations:** 1 Institute for Community Care and Health Equity, Chung-Ang University, Seoul, Republic of Korea; 2 Institute of Health and Environment, Seoul National University, Seoul, Republic of Korea; 3 Red Cross College of Nursing, Chung-Ang University, 84 Heukseok-Ro, Dongjak-Gu, Seoul, 06974 Republic of Korea; 4 Graduate School of Public Health, Seoul National University, Seoul, Republic of Korea; 5 Department of Industrial and Systems Engineering, Korea Advanced Institute of Science and Technology, Daejeon, Republic of Korea; 6 Graduate School of Science and Technology Policy, Korea Advanced Institute of Science and Technology, Daejeon, Republic of Korea

**Keywords:** Community Health Nursing, Home Health Nursing, Visiting Health Services, Aged; Case Management, Referral and Consultation, Mobile Applications, Artificial Intelligence, Information Systems

## Abstract

**Background:**

The role of visiting health services has been proven to be effective in promoting the health of older populations. Hence, developing a web system for nurses may help improve the quality of visiting health services for community-dwelling frail older adults. This study was conducted to develop a web application that reflects the needs of visiting nurses.

**Methods:**

Visiting nurses of public health centers and community centers in South Korea participated in the design and evaluation process. Six nurses took part in the focus group interviews, and 21 visiting nurses and community center managers participated in the satisfaction evaluation. Focus group interviews were conducted to identify the needs of visiting nurses with respect to system function. Based on the findings, a web application that can support the effective delivery of home visiting services in the community was developed. An artificial intelligence (AI) algorithm was also developed to recommend health and welfare services according to each patient’s health status. After development, a structured survey was conducted to evaluate user satisfaction with system features using Kano’s model.

**Results:**

The new system can be used with mobile devices to increase the mobility of visiting nurses. The system includes 13 features that support the management of patient data and enhance the efficiency of visiting services (e.g., map, navigation, scheduler, protocol archives, professional advice, and online case conferencing). The user satisfaction survey revealed that nurses showed high satisfaction with the system. Among all features, the nurses were most satisfied with the care plan, which included AI-based recommendations for community referral.

**Conclusions:**

The system developed from the study has attractive features for visiting nurses and supports their essential tasks. The system can help with effective case management for older adults requiring in-home care and reduce nurses' workload. It can also improve communication and networking between healthcare and long-term care institutions.

**Supplementary Information:**

The online version contains supplementary material available at 10.1186/s12913-023-10434-6.

## Background

As the burden of care increases with an aging population, the demand for home-and community-based care amplifies. The role of visiting nursing services is particularly important for improving health equity because they can identify the individual circumstances of vulnerable older adults and connect them with the required health and social care institutions in the community [1]. They can provide customized care by making shared decisions with patients over a long period of time, which can positively affect patients' health [2]. Despite the differences in healthcare systems, the positive effects of home visits on enhancing functional independence and health-promoting behaviors, and reducing hospital admissions among older adults have been reported in Western countries [3–5].

Recently, the integration of Information and Communications Technology (ICT) with healthcare services has received increasing attention for improving healthcare services. In 2012, the World Health Organization (WHO) published the National eHealth Strategy Toolkit to develop a national action plan for ICT-based health policy formulation, monitoring, and evaluation strategies [6]. Previous research showed that the use of ICT in healthcare services can improve access to and quality of services, promote patient-centered care, and enhance efficiency in decision-making and communication between care providers [7, 8]. Although the use of ICT is less common in home-care settings, attempts have been made to develop web-based systems [9] or software [10] to assist in patient assessment, data collection, and planning interventions. Moreover, the implementation of mobile technologies has diversified the service areas and functions of home care services [11].

In South Korea, public health centers provide multidisciplinary visiting healthcare services through nurses, doctors, and therapists based on the Regional Health Act [12]. These services aim to raise public health awareness, prevent diseases, and improve the self-management of chronic conditions of community residents, especially vulnerable populations [13]. Visiting healthcare services provide a wide range of primary care services, which differ from those of hospital-based services that focus on disease treatment and acute care. Visiting nurses provide individual health interventions for high-risk groups (e.g., frail older adults living alone or individuals with multiple care needs) and group education, conduct public health campaigns for local residents, and link resources in the community [14]. Visiting healthcare services in Korea have been shown to be effective in the management of hypertension [15], and improvement of frailty and depressive symptoms in older adults [16]. As home-visiting healthcare services have expanded, various projects reflecting regional differences in population characteristics and care needs have been developed.

Visiting nurses at public health centers use an integrated public healthcare information system (PHIS) developed by the Ministry of Health and Welfare of Korea. PHIS have enhanced work efficiency and reduced costs by implementing electronic medical record systems in public health centers [17]. However, the system is not without limitations despite several improvements since 1994. It has been reported that it is difficult to share information with medical institutions in the community, and that improvements are needed to use the system in mobile services [17]. Web-based systems using mobile devices have the potential to facilitate home care services, given the unique work environment of visiting nurses who spend a high proportion of work time driving to the homes of patients [18]. Moreover, they need to make autonomous decisions in accordance with patient needs and effective work schedules to provide patient-centered and timely care [19].

This study was conducted to develop a new web application (“CARE-Net”) to enhance the quality and efficiency of visiting healthcare services. The objective of the study was to develop a platform that supports the care management of visiting nurses and strengthens the linkage and cooperation between public health centers and healthcare organizations in the community. The platform is intended for use with wireless devices, including tablet PCs. The results are expected to be used as basic data for the development of smart visiting healthcare services using ICT.

## Methods

### Study context

In 2021, there were 258 public health centers in every city or district (“gun" or "gu”) in Korea. In some regions, particularly rural areas, 1,342 branches of public health centers and 1,904 public health posts have been operated [20]. All visiting nurses working in public health centers used the web-based PHIS to record patients’ health status and interventions for each home visit. However, the PHIS was intranet-based used by public health center employees with desktop computers at the centers.

### Participants

The main users of the CARE-Net system are visiting nurses working in public health centers. Due to the scarcity of previous research, it is important to understand the experience of visiting nurses working in the community. Therefore, home-visiting nurses working in public health centers in Korea participated in the design and evaluation process. The nurses were recruited from public health centers using purposive sampling methods, to select information-rich cases for our research context [21]. The potential participants were recruited with the cooperation of local governments and center managers. The nurses with experience in visiting health services, who agreed to participate, were included in the study.

Focus group interviews were conducted with six nurses using a semi-structured questionnaire to understand the nature of their work and their needs regarding the web-based work system. These nurses were from centers in Seoul, the capital city of Korea. After the system was developed, 21 visiting nurses and managers from community centers in Chuncheon City participated in the evaluation process. These centers were located in both urban and rural areas. We invited nurses from all centers in the City and those who agreed to participate in research were included. They attended training sessions on the functions of the CARE-Net system before participating in the evaluation survey.

### System development

**Fig. 1:**
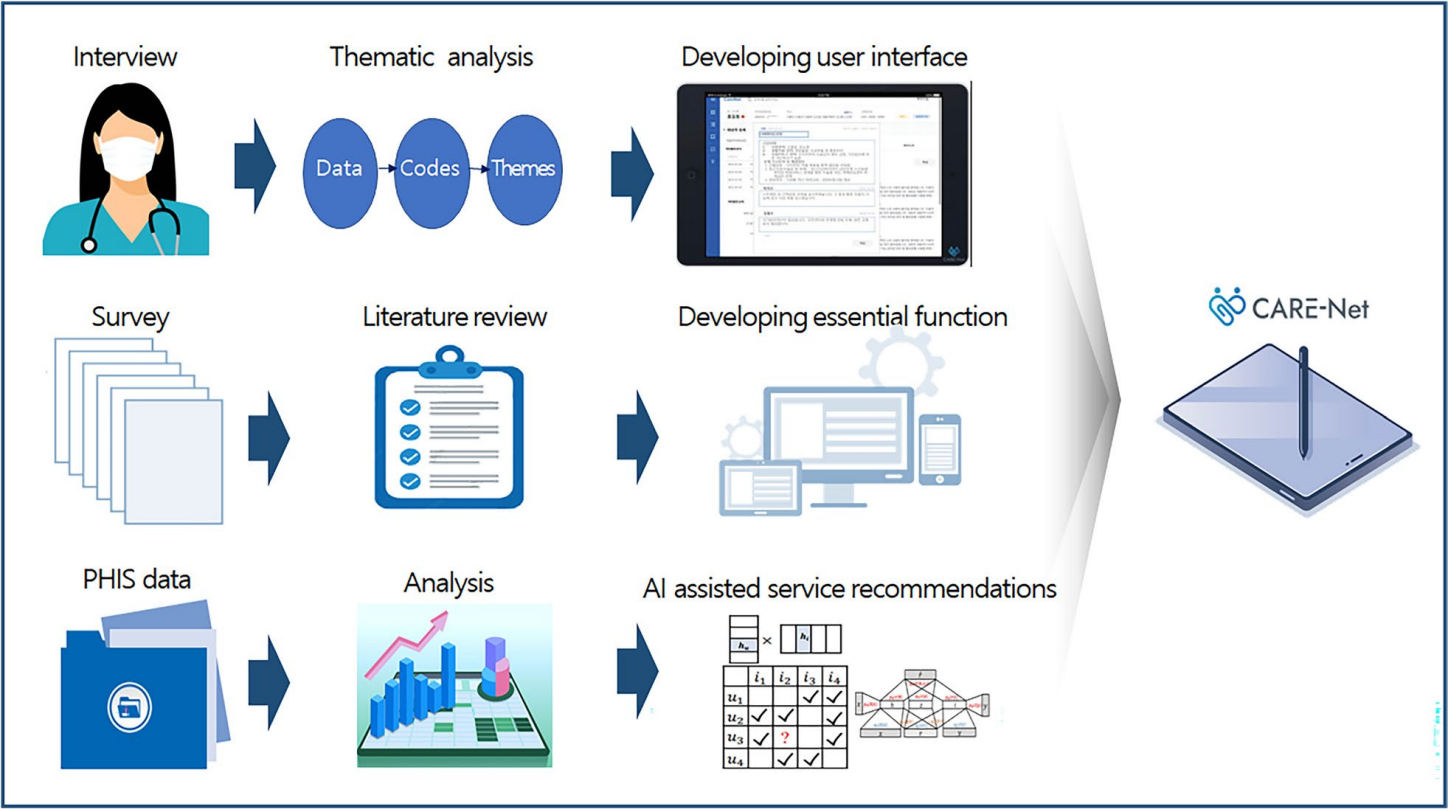
The process of developing the system

The system development process is illustrated in Fig. 1. We reviewed research articles and related documents (e.g., work manuals and government reports) on visiting nurse services and health information systems in Korea and other countries. We conducted interviews with visiting nurses to identify their work experiences and needs regarding the features of the system to help them provide effective care to patients in the community. The workflow for visiting healthcare services was identified. The patterns of current system utilization and requirement of system features were defined based on the interviews. The PHIS data were analyzed to develop an artificial intelligence (AI) algorithm for recommending services linked to health and welfare [22]. The AI-based algorithm in the system can assist nurses by recommending appropriate service connections based on previous work by senior nurses. Based on these findings, we developed an integrative web application called CARE-Net that can support the effective delivery of home-visiting services in the community.

Additionally, in the development of our platform, we prioritized data security and ethical considerations. To protect patient data, we implemented multiple security layers, including encrypted data transmission, secure server hosting, and restricted access controls to ensure that only authorized personnel access sensitive information. Regular security audits and updates are standard practice to uphold these high-security standards. Furthermore, we required all potential users to sign a consent agreement, acknowledging their understanding and commitment to data privacy and ethical handling of patient information. This agreement detailed their responsibilities for maintaining confidentiality and ethical standards. For patient data, our platform features an electronic informed consent process, enabling patients to control the extent and recipients of their shared data.

### Evaluation of user satisfaction

We used the Kano model to evaluate user satisfaction with CARE-Net features [23]. Although the Kano model was initially developed to enhance the quality of manufactured goods, it has also been applied to healthcare services [24–26]. The model employs a questionnaire to categorize each quality attribute. Questionnaires were developed based on whether a feature was available (functional form) or not (dysfunctional form). An evaluation table (Table 1) was used to categorize each attribute into one of five quality dimensions: (O) one-dimensional, (A) attractive, (M) must-be, (I) indifferent, and (R) reverse. A questionable result (Q) denotes the uncertainty regarding whether the respondent correctly comprehended the question. The six quality attributes are described as follows:

i. (O) One-dimensional quality: This quality factor has a linear relationship with customer satisfaction, which is satisfied when sufficient and dissatisfied when insufficient.
ii. (A) Attractive quality: This quality attribute increases customer satisfaction when sufficient but does not cause dissatisfaction when insufficient.
iii. (M) Must-be quality: This quality attribute is taken for granted; improving it does not make a difference to satisfaction, and its lack leads to dissatisfaction.
iv. (I) Indifferent quality: This quality attribute does not affect customer satisfaction.
v. (R) Reverse quality: This quality factor, if present, lowers customer satisfaction.
vi. (Q) Questionable result: erroneous response due to misunderstanding of questions.

**Table 1:** KANO evaluation table

	A feature is not available (Dysfunctional form of question)					
		I like it that way	It must be that way	I am neutral	I can live it that way	I dislike it that way
A feature is available (Functional form of question)	I like it that way	Q	A	A	A	Q
	It must be that way	R	I	I	I	M
	I am neutral	R	I	I	I	M
	I can live it that way	R	I	I	I	M
	I dislike it that way	R	R	R	R	Q

^a^*A* Attractive, *O* One-dimensional, *M* Must be, *Q* Questionable, *R* Reverse, *I* Indifferent

### Data analysis

Audio recordings from semi-structured interviews were transcribed in Korean. Thematic analysis was conducted to identify themes related to nurses’ work experience and need for a web-based work system [27]. The transcripts were read several times to understand the implications of the nurses’ experiences and system requirements, and were coded for themes. The research team held regular meetings to compare and resolve differences in the researchers’ coding. The results were validated through discussions with the researchers and advice from nursing researchers and visiting nurses.

After the development of the system, the participants were included in a pilot project and completed the survey. Data from the user satisfaction survey were descriptively analyzed based on the Kano model. Out of the responses received from 21 visiting nurses in Chuncheon City, a total of 15 were considered for analysis, as data from 6 participants were incomplete in the satisfaction survey. All statistical analyses of the satisfaction survey were performed using SAS 9.3 (SAS Institute Inc., Cary, NC, USA).

## Results

### Conceptualization of the system

The new “CARE-Net” system was designed for effective patient health management considering the characteristics of home visiting services. Until now, visiting nurses could only use desktop computers in public health centers after home visits, which increased their workload. To facilitate higher mobility, the new web-based system is intended to be used with a tablet PC or mobile device. Visiting nurses can review or record information, use online educational materials in the patients’ homes or while commuting between homes. The main users of the existing PHIS were visiting nurses at public health centers. However, we expanded the user categories by including healthcare workers in public health centers (e.g., doctors, social workers, nutritionists, and physical therapists) and other institutions in the community (e.g., local primary care doctors and welfare centers).

### Workflow of the visiting healthcare services

The workflow of visiting healthcare services was identified as follows: 1) Patient registration, 2) Home visit, 3) Initial evaluation of health conditions and socioeconomic status, 4) Case management (establishment of care plan and provision of nursing intervention), 5) Nursing record, 6) (If necessary) Patient referral or linkage with related health and care institutions in the community, 7) End of visit. The PHIS user manual used in public health centers was analyzed to identify the main system functions related to each work stage. We found that visiting nurses need to establish care plans for each patient and work with multidisciplinary professionals to ensure comprehensive case management. On some occasions, the public health center refers the patient to other healthcare institutions for further support. However, the PHIS lacks the ability to review or document information related to each patient’s case management. Moreover, it was not possible to share information with other healthcare or welfare institutions in the community through the system during patient referral. Therefore, we decided that features that support documentation of the care plan and information sharing with related institutions were necessary for the new system.

### Focus group interviews

Based on the interviews, we analyzed the difficulties in using the PHIS and provided suggestions for improvement according to the workflow (Table 2). Before the visit, the nurses registered the patients, made reservations, and collected basic patient information. Participants reported that the current work system was not efficient in obtaining patient information because they could not access other social databases through the system. Additionally, it was difficult to organize, search for, and register patient information because visiting healthcare services often target economically disadvantaged families and the current system collected household data. However, nurses reported the need to obtain individual patient data for effective case management.

**Table 2:** Difficulties in using the work system and suggestions for improvement derived from interviews

Step	Work flow	Difficulties in using the PHIS	Suggestions for improvement
Pre-visit	Registration	- Difficulty in obtaining a patient’s information due to lack of access to other social databases - Difficult to organize, search for, and register the patient’s information because the current system is based on household information, rather than individual data No place to write patient information such as name and phone number before registration	- Needs a system that focuses on each patient’s individual data Requires a user interface to write patient information prior to registration
Pre-visit During visit	Visit reservation	- No place to write a record of calls or to reschedule - No function for sorting patient lists - Difficult to search for patient information by their characteristics, such as address	- Requires an interface to record phone calls - Requires a user interface that makes it easy to reschedule reservations (e.g., calendar) - Requires a user interface to organize patient lists according to visit date and patient characteristics (e.g., birth date, address)
	Collecting information before visit	- No place to write information collected prior to the visit	- Requires a user interface to write patient information immediately
	Home visit	- Time consuming to coordinate complex schedules and movement - Difficult to carry heavy bag with medical supplies, education materials, etc - Difficult to provide health education due to the lack of time and proper educational materials	- Requires a platform that can be used with wireless devices (tablet PC) - Requires a user interface to check schedules and find efficient travel routes (e.g., map or navigation function) - Need to include various forms of patient educational materials on the platform for nurses
During visit Post-visit	Health assessment	- Too many questionnaires about health behaviors - Insufficient assessment of medical history, such as medication - Some assessment forms have not been updated or do not match with actual work	- Need to revise the assessment forms for visiting nurses
	Nursing plan	- Lack of a nursing plan - Lack of interaction with other health care professionals	- Need to develop content to assist with making nursing plans - Requires a user interface to record nursing plans - Requires a user interface that facilitates discussion with other health care professionals to establish a care plan for each patient
	Nursing intervention	- Setting the visit cycle according to the group classification - Click and write whether nursing is performed on the fixed form	- Need to set the visit cycle according to the nurse’s diagnosis - Requires a user interface that can be written in narrative form
	Nursing record	- Cannot write the results of nursing care while at the visit location - Too much time spent recording on computer system after returning to the office - Cannot upload pictures or specific documents related to patients - Difficult to analyze the basic health status of patients	- Requires a user interface that can be used on a mobile device - Need to consider convenience of recording notes at the place of visit - Need a function that allows for uploading or downloading files on the system - Requires a function that provides statistical analysis based on patient information (e.g., basic tables and graphs)
Post-visit	Other resource linkage	- Relying on nurse’s personal information in linking community resources - Taking the time to send a service request form by fax or mail - Difficult to receive a response when requesting results	- Need to provide information on other medical and social resources - Need to recommend possible linking services - Requires a user interface to send and receive service requests and responses
	End of visit	- Duplication of work and difficulty in reusing existing patient data due to repeated registration at end of visit - Visit ends when a patient receives national long-term care benefits although the older patients still need care in community	- Need to reuse patient data when nursing visits resume, even after termination of nursing visits Nurses need to coordinate and link necessary health and social care services in communities for frail older patients

Moreover, the previous work system did not record detailed patient information before registration or results from telephone interviews. The need for a user interface (UI) that allows convenient scheduling and organization of patient lists according to the visit date and patient characteristics was identified. Some nurses suggested that including an online calendar in the application would assist in effective scheduling. They also wanted to record patient information instantly in the work system.

*“I wish I could click on it and see the list. [When I click] my region and my name, those who are in their 60s, appear [in the system], but it would be nice if the list, not the number of household members, was also displayed. (…) I have to count how many people I have, but I hope my performance is shown with my name in the system by month. I feel very uncomfortable when counting this for monthly performance statistics.”*

Regarding the home-visit phase, nurses found it difficult to coordinate complex schedules with multiple patients and carry heavy bags of medical supplies and educational materials. They felt there was a lack of time to provide health education in patients’ homes. Therefore, they wanted a platform that could be used with wireless devices. They wanted various educational materials (e.g., documents, pictures, and videos) for patients to be included in the system. Moreover, they wanted to check the schedule, patient addresses, and maps (navigation functions) through the system.

*“I carry a blood pressure monitor, a diabetes monitor, an anemia monitor, and a cholesterol monitor, when necessary, in my bag. Cancer patients need something like porridge, and I also need a tape measure, a hand dynamometer, a weight scale, handbooks, and these days everyone has to wear a mask as well. And hand sanitizer (…) I also carry a laptop with me. It's a bit big and heavy, it's old. I carry a laptop and some device for connecting Wi-Fi, and I also carry a mat to put under the weight scale, so I really have to carry a lot.”*

In terms of health assessment, the nurses reported the burden of paperwork related to patient health survey forms. They wanted simplified forms that included the essential content. They also felt that the questionnaires did not sufficiently include items related to patients’ medical histories, and some assessment forms had not been updated to reflect the actual workflow. In terms of nursing plan, the participants reported the need for functions to support its establishment and recording. Additionally, they felt the need for more interactions with other healthcare professionals through the system. Moreover, they wanted to establish a visit plan according to their own diagnosis and record detailed information in a narrative form. They typically write detailed narratives related to patients and interventions in the "service record" because it is the only function that allows narrative writing in the system.

*“Questions like ‘do you walk for more than 10 minutes?’ Mostly we ask a lot of questions. ‘Do you engage in an intensive exercise?’ or ‘do you exercise vigorously?’, like this. It would be nice if I could ask just one or two simple questions about exercise.”*

*“I spend a lot of time writing service records. I write long and in detail on the service record. When I meet a person a second or third time, I know that person and I simply check their safety. But when I first meet someone, I write down everything, from head to toe. Because it is not like hospitals where I see people lying down every day, and if I saw a person today, no matter how soon I meet them, I'll meet them again in a week. You can even see them one or two months later.”*

After the visit, nurses recorded the results of the home visits and nursing interventions. Currently, nurses spend a considerable time recording this information on computer systems after returning to office. They required a UI based on a mobile device that could be used during home visits or while commuting. Additionally, they required a function to upload pictures or specific documents related to the patients. They reported the need for a function that provides statistical analysis based on patient information (e.g., basic tables and graphs).

*“You cannot upload an X-ray picture of an individual and the progress of that person in the system because I cannot leave it as a public document. I felt a bit sorry about that. When we have a medical certificate or something like an X-ray picture, sometimes we want to upload those files.”*

When necessary, nurses refer patients to community resources such as primary health clinics and welfare centers. Currently, they must rely on personal efforts to obtain information and cooperate with community institutions. It takes time to send a service request by fax or mail and it is difficult to receive responses from institutions. They suggested that the system should provide information on various medical and social resources in the community. Moreover, they wanted new features that could help with case management and link with other institutions in the community.

*“We just have to find the resources on our own. It would be good for the system to show those resources.”*

*“This is not something that can be solved all at once, so I explain the situation to the person in charge. But there are a lot of patients who are in ambiguous situations. There are many people who do not meet the standard but still need care. (…) It's not just by calling again, I should call several times. It takes a bit of time when we prepare all the required documents, such as a request form, and send them as official documents to outside institutions. It takes a lot of time to link one person, but no matter how much time and effort into it, we cannot leave a record of everything about them.”*

The visit typically ends when the patient moves to another region, receives national long-term care benefits, or dies. However, some patients return to the center to receive services several years after the end of visit. Participants reported difficulties in reusing patient data when the patient was reregistered for the program. The registration process needed to be repeated, which led to duplicate data. They felt the need to reuse patient data when visits resumed. Additionally, they felt the importance of coordinating and linking appropriate health and social care services for frail older patients when the visit ended because those who receive long-term care insurance services have various care needs.

*“For one or two years after we register a person, I won’t have to meet the patient if there is no health problem, and the visit ends. It kept going like this. Then, after another 2 to 3 years, we might meet them again as they get older; then, we register them again.”*

*“If the address remains...You can see where the patient went only if social workers wrote it down in the database. (…) Sometimes, it seems that patients went to a long-term care hospital located nearby. However, I cannot search for it unless it was recorded [in the system].”*

### System features

**Fig. 2:**
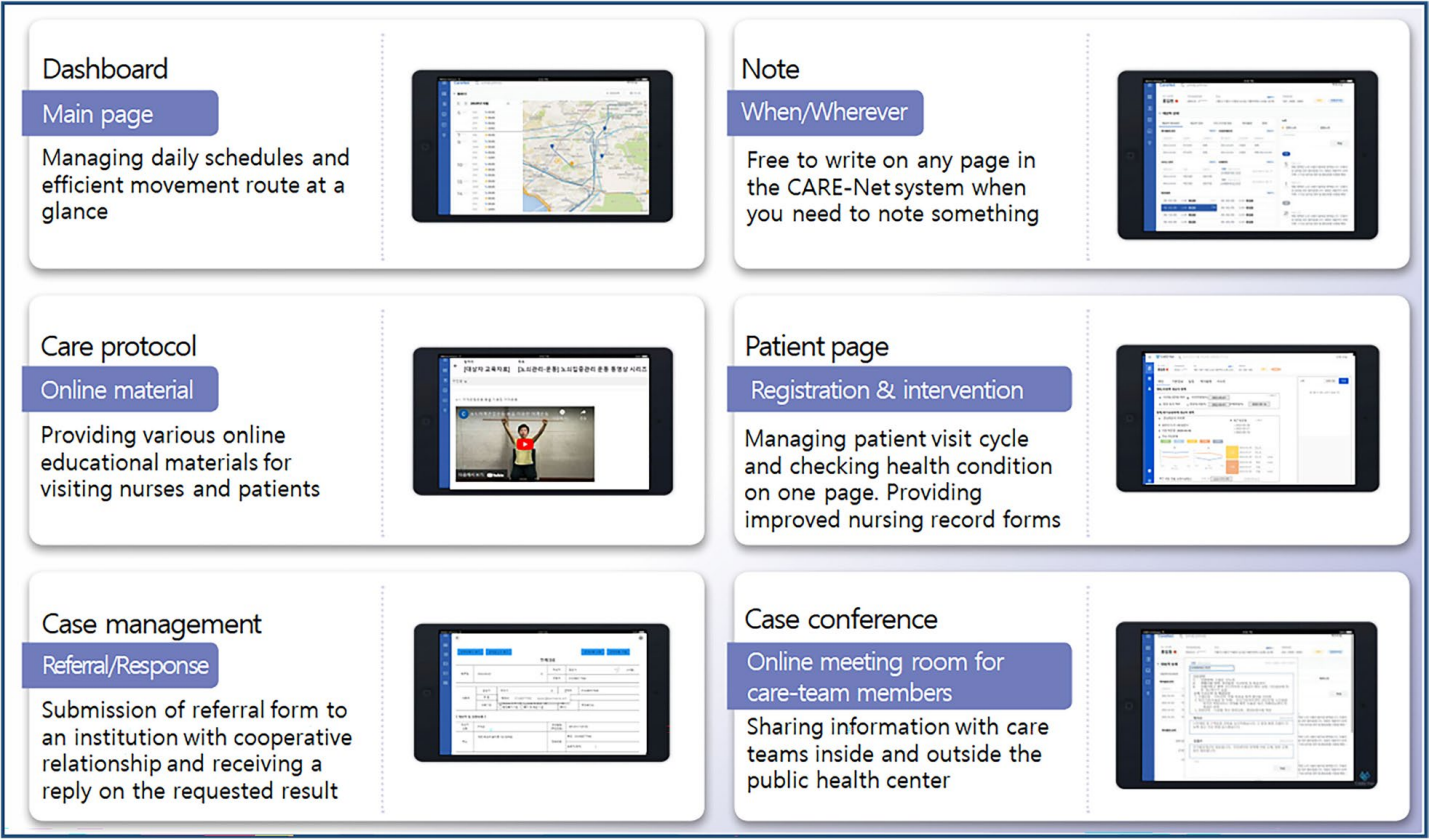
Example images and descriptions of main functions in the system

Considering the difficulties in using PHIS and the suggestions for improvement derived from interviews, this study developed 13 system features (Table 3). The key aspects of the system design and its features are shown in Fig. 2. The CARE-Net system, a web application, was developed to function on mobile devices, such as tablets and smartphones. CARE-Net consists of two UI parts: one for patient data input and output and one for nurses' convenience during the visit.

**Table 3:** User interface and various features in CARE-Net system

Category	Description
**Change of system**	
Mobile-based system	CARE-Net utilizes a tablet to write down patient information, conduct case conferences, and connect services to partner organizations while visiting and on the go
Basic information input unit	CARE-Net converts the basic information input unit from the family to the individual patient
**Changes of user interfaces for visiting nurse**	
Dashboard	UI for visiting nurse's schedules, accessing patient’s records, checking visiting location, and navigation on one screen
Patient list	UI for that includes patient's name, gender, address, contact information, registration date, last visit date, and registration status on one screen
Main screen	UI for checking the phone call history of pre-registration subjects, classifying service group, and setting visit cycle according to the nurse's clinical judgment. In addition, it provides a chart of the patient's recent major health problems and recent health status
Basic information	UI for recording patient’s basic information, such as name, address, and date of birth. Pre-registration is available only with name and contact information for efficient patient management before official registration
Scheduler for each patient	UI for setting the schedule for home visits and phone visits, and the information is linked together with the calendar and navigation
Care plan	UI that can set nursing plan and goals according to each patient's major health problems based on the nurse's clinical judgment. It can also create an integrated nursing plan that includes an AI-based linked service recommendation system
Nursing record	Nursing record forms were developed to simplify the low-priority questionnaires in the clinical field and enable nurses to plan visits according to their clinical judgment. Moreover, detailed instructions are provided on the nursing record form to assist low-skilled nurses with performing therapeutic interventions according to standardized guidelines
Case management	UI that supports case conferences with the integrated health care team, makes online referrals, and receives responses from affiliated institutions. When writing a new post on this UI, a notification is provided to the designated people through Kakao Message
Scheduler	UI for scheduling nurses’ daily work. It is possible to record additional schedules such as official meetings as well as home and phone visit schedules for each nurse. In addition, the to-do list provides a summary of each day's work so that nurses can see it at a glance
Map and Navigation	UI that displays the visit locations (as spots on a map) in the schedule for the current day and provides navigation from the current location to next when the spots are clicked
Care protocol	UI that provides various manuals and educational materials for using the CARE-Net system, nursing intervention, and linking medical and social welfare services
Care coach	UI where external expert advisory teams (university professors, nurse practitioners, senior nurses, etc.) provide constant advice on patient care and work. When questions and answers are registered, a notification is provided to the designated people through Kakao Message
**Added user interfaces for linked institute**	
List of patients requesting linkage	UI displaying the list of patients for whom the visiting nurse has requested a health and welfare services referral. When a new referral request is registered, a notice is sent via Kakao message
List of patients to be linked	UI showing a list of patients who have accepted the request for health and welfare service referrals from the visiting nurse. If the request is accepted, all patient records, including care-plan, nursing records, case management, and care-coach, can be viewed. However, the affiliated institution only has the authority to create and edit the case management UI

The patient data input/output system includes sub-UIs centered on patient list. The list provides brief personal information, such as name, gender, and address. When a patient's name is clicked, it links to sub-UIs, including the main screen, basic information, scheduler, care plan, case management, and nursing records for the patient. The patient's main screen provides records of the recent nursing visits and health status. The care plan is a UI that helps create a specific nursing visit plan based on health problems automatically derived from nursing records. In particular, the care plan is equipped with an AI-based linked service recommendation system. An algorithm for recommending services within and outside public health centers has been developed to activate linkages and cooperation within the community. Based on patient information recorded in the system, the algorithm recommends various programs and services implemented by public health centers, local clinics, welfare centers, and long-term care centers in the community.

The case management UI helps users create online case conferences with multidisciplinary teams and send online documents for referral and responses to medical, social welfare, and administrative agencies. The UIs for visiting nursing support provides features including maps and navigation, calendars and schedulers, protocol archives, professional nursing advice, and community diagnosis. The UI of protocol archives, called the care protocol, provides various manuals and educational materials for nursing intervention and medical and social welfare services. Through the care coach UI, questions can be asked to a professional coaching team composed of multidisciplinary professors and senior visiting nurses during patient care and answers can be obtained online. Additionally, the system has a feature for issuing alarms through mobile phone message. CARE-Net also supports an online tool for nurses’ community diagnosis and statistics based on patient information.

### Examples of the system usage

CARE-Net can be used to manage frailty and chronic diseases in older adults and for community networking through mutual referrals with health and social care institutions (Supplementary file 1). The system includes a tool that automatically calculates a score to determine frailty status. Users can check a patient’s frailty score using the main dashboard. It aims to assist nurses in identifying frail elderly patients and establishing care plans for them. In chronic disease management, patients with uncontrolled hypertension and diabetes are targets of service provision. When local primary care clinics refer patients to visiting nurses, nurses can conduct health surveys, establish care plans, and conduct home visits using CARE-Net.

Furthermore, the referral system can be improved by using CARE-Net. In South Korea, older adults aged 65 years or above can receive services through the national long-term care (LTC) insurance after applying for a needs assessment conducted by the National Health Insurance Service (NHIS). Visiting nursing services at public health centers do not cover those who received LTC grades from the NHIS because public health centers focus on preventive services. In LTC institutions, visiting nursing services based on a doctor’s prescription are covered by national LTC insurance. Because many patients are unfamiliar with these policies, nurses at public health centers can refer patients with LTC grades to LTC institutions.

### Evaluation of the system

Satisfaction with the system’s features was surveyed among nurses participating in the pilot project. As shown in Table 4, more than half of the nurses had high satisfaction with all features. Especially, the "care plan" equipped with AI-based linked service recommendations showed the highest satisfaction. Table 5 lists the service quality dimensions based on the Kano model for each CARE-Net system feature. All the functions were evaluated as one-dimensional quality features. The "map and navigation" feature showed the same response rate for one-dimensional and attractive qualities. In addition, the ‘case management’ feature, which enables one to make online referrals and support case conferences, also recorded a higher attractive score compared to other features. These results indicated a strong preference among the nurses for features that facilitate efficient home visits and effective communication and management of patient care. However, other features, including the mobile-based system, schedulers, care protocols, and care coaches, were identified as natural quality factors, integral to the nurses' work. It was observed that these components, while necessary, did not significantly enhance overall satisfaction.

**Table 4:** Simple satisfaction results of CARE-Net system features (*N* = 15)

Question	Positive response, n (%)^*^
Mobile-based system	10 (66.7)
Dashboard	8 (53.3)
Patient list	10 (66.7)
Main screen	10 (66.7)
Basic information	11 (73.3)
Scheduler for each patient	11 (73.3)
Care plan	14 (93.3)
Case management	12 (80.0)
Nursing record	9 (60.0)
Scheduler	12 (80.0)
Map and navigation	11 (73.3)
Care protocol	11 (73.3)
Care coach	12 (80.0)

^a^The proportion of the response of ‘I like it that way’ and ‘It must be that way’ in the functional form of questions

**Table 5:** Quality features of CARE-Net system features based on Kano’s model (*N* = 15)

	A	M	O	I	R	Q
Mobile-based system	3 (20.0)	3 (20.0)	7 (46.7)	2 (13.3)	0 (0.0)	0 (0.0)
Dashboard	3 (20.0)	3 (20.0)	5 (33.3)	4 (26.7)	0 (0.0)	0 (0.0)
Patient list	1 (6.7)	3 (20.0)	9 (60.0)	1 (6.7)	0 (0.0)	1 (6.7)
Main	1 (6.7)	2 (13.3)	9 (60.0)	2 (13.3)	1 (6.7)	0 (0.0)
Basic information	1 (6.7)	1 (6.7)	10 (66.7)	2 (13.3)	1 (6.7)	0 (0.0)
Scheduler for each patient	2 (13.3)	1 (6.7)	9 (60.0)	3 (20.0)	0 (0.0)	0 (0.0)
Care plan	4 (26.7)	0 (0.0)	9 (60.0)	1 (6.7)	0 (0.0)	0 (0.0)
Case management	5 (33.3)	1 (6.7)	7 (46.7)	1 (6.7)	1 (6.7)	0 (0.0)
Nursing record	2 (13.3)	3 (20.0)	7 (46.7)	2 (13.3)	1 (6.7)	0 (0.0)
Scheduler	4 (26.7)	0 (0.0)	7 (46.7)	3 (20.0)	0 (0.0)	1 (6.7)
Map and navigation	5 (33.3)	2 (13.3)	5 (33.3)	2 (13.3)	0 (0.0)	1 (6.7)
Care protocol	4 (26.7)	2 (13.3)	6 (40.0)	2 (13.3)	0 (0.0)	1 (6.7)
Care coach	4 (26.7)	0 (0.0)	7 (46.7)	2 (13.3)	0 (0.0)	1 (6.7)

^a^*A* Attractive, *M* Must be, *O* One-dimensional, *I* Indifferent, *R* Reverse, *Q* Questionable result

## Discussion

In this study, we developed a new mobile healthcare system that addresses the needs of visiting nurses. We focused on the three key requirements of mobility, timeliness, and connectivity in the development of CARE-Net. It is a web application through which nurses can input and manage patient data onsite through mobile devices. An online referral and response system is implemented in it to enable timely referrals and connections between medical, social welfare, and administrative agencies. Furthermore, various features have been developed to help visiting nurses, including maps, navigation, schedulers, protocol archives, professional nursing advice, and online case conferences. In the evaluation of system satisfaction, most nurses showed high satisfaction. In particular, the care plan with AI-based connected service recommendation showed the highest satisfaction among all features.

Mobile health (mHealth) technology combines mobile communication and computing in a handheld device, enabling mobile computing in the field. Mobile applications have been developed for, and used by, medical professionals in various fields, such as hospital information systems (HIS), disease diagnosis, and clinical communication [28]. The CARE-Net is an information system with a format similar to that of HIS. It has been designed to record nursing data online during home visits and to provide access to previous nursing records. However, developed as a web application, CARE-Net overcomes the limitations of the PHIS [17].

CARE-Net enables online conversations with colleagues and provision of multimedia texts. Unlike traditional clinical applications, it is focused on multidisciplinary collaboration with experts in various disciplines. Active communication with experts outside the center informs decision-making, leading to better patient outcomes. Previous studies have shown that a multidisciplinary approach effectively improves patients’ frailty status, health, and quality of life [29, 30].

Furthermore, the AI-based recommendation algorithm developed in this study can facilitate effective linkage services by providing lists of available community resources and care policies. A previous study reported that tasks linked to community resources, such as social welfare and clinical services, showed the lowest confidence and job performance among nurses [31]. In the satisfaction survey results, the positive response rate for the care plan with AI-based recommendations was the highest among all CARE-Net features. Additionally, the results revealed the difficulties in connecting with community resources for visiting nurses working in public health centers.

System requirements have been developed based on individual and focus group interviews with visiting nurses working in local communities. To develop an effective work system, it is crucial to meet the needs of system users. Qualitative methods provide effective ways to engage stakeholders in health research, such that a greater perspective is gained from the in-depth experiences and opinions of the participants [32]. Additionally, it allowed the involvement of the end-users throughout the development and evaluation processes. For instance, the need for a map function and the use of mobile devices reflect the nature of visiting care services that require high mobility. Moreover, nurses’ need for system functionality that supports case management and community linkages was also identified, leading to the development of our AI-based recommendation algorithm.

Based on Kano's quality attributes, all features of CARE-Net were evaluated as one-dimensional quality attributes. Only the map and navigation feature had the same score for attractive and one-dimensional quality attributes. A one-dimensional attribute is a performance requirement that users typically demand and expect from the proposed service [26]. These results suggest that the presence of most of the features was associated with maintaining user satisfaction, and that the absence of these features may lead to dissatisfaction. The attractive quality attribute has the most significant impact on user satisfaction. Its presence provides great satisfaction; however, satisfaction does not decrease if it is not met [23]. Therefore, attractive requirements differentiate our service from the others. These results demonstrated that the CARE-Net system successfully supports the essential tasks of visiting nurses and, at the same time, has the attractive features. Because nurses spend a substantial amount of time on patient visits, the map and navigation connected to the daily patient visit schedule may unexpectedly increase their satisfaction.

### Limitations

The CARE-Net system provides evidence for future research on smart healthcare systems for visiting services. However, this study has some limitations. Although the pilot project was conducted with the cooperation of city officials, the number of participants was limited because each center had only one nurse. Home visits by nurses may have been limited due to the effects of COVID-19. Considering the regional differences in work environments and care policies in Korea, further evaluation of the system with visiting nurses from different regions is needed.

## Conclusions

This study identified visiting nurses’ need for an effective ICT system that enhances the efficiency of visiting health services. The results of system evaluation suggest that the CARE-Net system meets their needs by providing features tailored to visiting services. This system could help in the effective case management of community-dwelling older adults. This would also reduce nurses' workload and enhance effective referrals between healthcare and LTC institutions.

### Supplementary Information

**Additional file 1: Supplementary Figure 1. **Examples of visiting nursing services using CARE-Net: Intensive frailty management program.  **Supplementary Figure 2.** Examples of visiting nursing services using CARE-Net: chronic disease management. **Supplementary Figure 3.** Examples of visiting nursing services using CARE-Net based on community networking and cooperation in a multidiscipline team. 

## Data Availability

The care-net described in this paper can be accessed at http://cn.summary-ai.com:8080/login, and you can download its application from the Google Play Store at https://bit.ly/CARE-Net. Access to CARE-Net is exclusively granted to users who have successfully completed a registration and approval process. Such restricted access is imperative, as CARE-Net includes sensitive patient data, inclusive of personal information, thereby necessitating rigorous adherence to data privacy and regulatory compliance protocols.

## Abbreviations

AI: Artificial intelligence
HIS: Hospital information systems
ICT: Information and Communications Technology
LTC: Long-term care
mHealth: Mobile health
NHIS: National Health Insurance Service
PHIS: Public healthcare information system
UI: User interface
WHO: World Health Organization
