# Supplementary material for: Development of a web-based care networking system to support visiting healthcare professionals in the community
Source: BMC Health Serv Res. 2023 Dec 16;23:1427. doi: 10.1186/s12913-023-10434-6 (PMC10725602; doi:10.1186/s12913-023-10434-6)

Supplementary materials

Supplementary Figure 1. Examples of visiting nursing services using CARE-Net: Intensive frailty management program

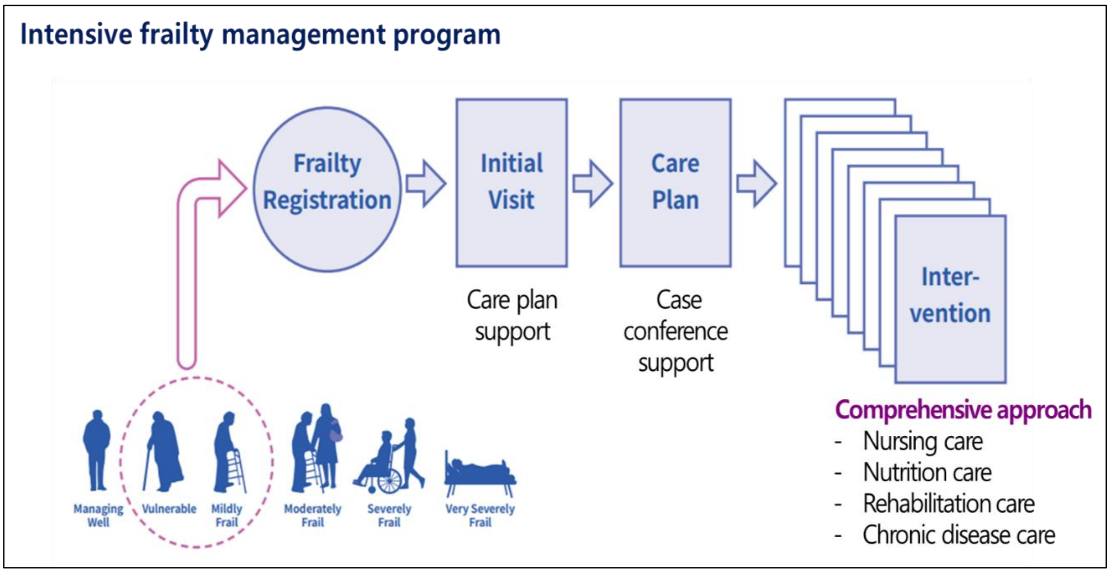

Supplementary Figure 2. Examples of visiting nursing services using CARE-Net: chronic disease management.

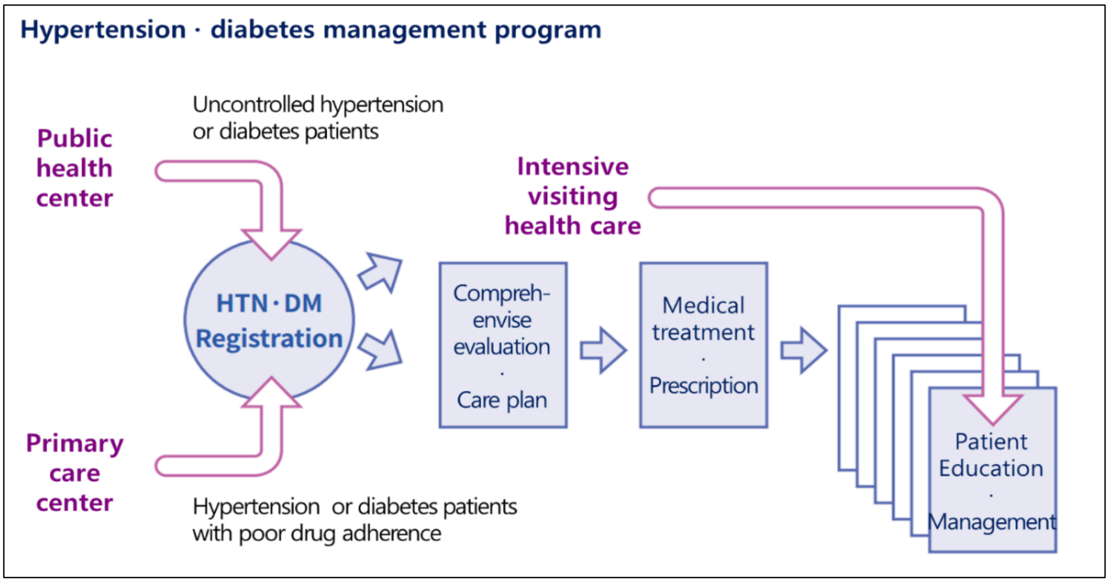

Supplementary Figure 3. Examples of visiting nursing services using CARE-Net based on community networking and cooperation in a multidiscipline team.

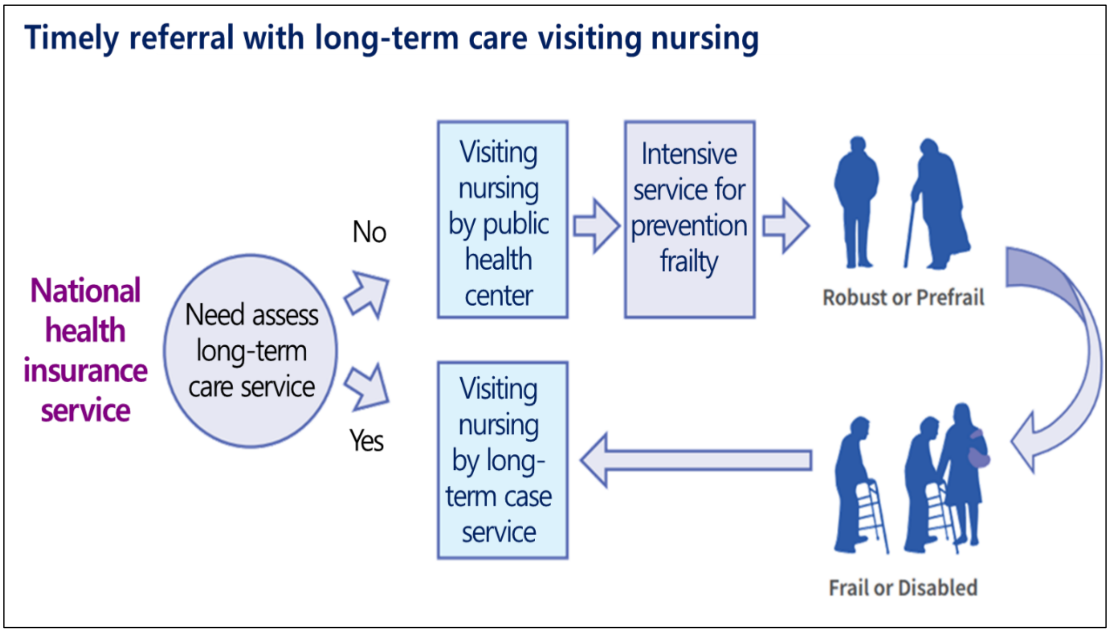

Supplement: Supplementary file 1 — Additional file 1: Supplementary Figure 1. Examples of visiting nursing services using CARE-Net: Intensive frailty management program. Supplementary Figure 2. Examples of visiting nursing services using CARE-Net: chronic disease management. Supplementary Figure 3. Examples of visiting nursing services using CARE-Net based on community networking and cooperation in a multidiscipline team. [file 12913_2023_10434_MOESM1_ESM.pdf]
